# Perivascular epithelioid cell tumour (PEComa) of the ligamentum teres hepatis, a rare and mobile tumour presentation

**DOI:** 10.1111/ans.19418

**Published:** 2025-02-28

**Authors:** Jacques‐Emmanuel Saadoun, Eddy Traversari, Hélène Meillat, Jérôme Guiramand

**Affiliations:** ^1^ Department of Surgical Oncology Institut Paoli Calmettes Marseille France; ^2^ Department of Surgical Oncology Institut Paoli Calmettes Marseille France

A 25‐year‐old man with no medical history consulted his general practitioner following self‐palpation of a mobile abdominal mass. Clinical examination demonstrated the highly mobile nature of the mass, as can be seen in the video. He did not present any digestive symptoms. Computed tomography showed a large 11 cm heterogeneous mass, partially calcified and necrotic, located in the left flank. On computed tomography (CT) scan, the mass appeared to originate either from the mesentery or a small bowel loop. Significant contrast enhancement was observed, including during the portal phase, with a necrotic core. Work‐up was completed by MRI, which showed a mass with necrotic core that had moved to the right flank. MRI suggested a mesenteric origin for the mass. Pre‐contrast, the mass borders had an isointense T1 signal, which showed variable enhancement of the tumour post‐contrast. The mass's center exhibited mixed composition, including a 3 cm region of avascular necrosis. This heterogeneity suggested a desmoid tumour in the peritoneal cavity. An ultrasound‐guided biopsy was performed, and histological analysis supported the diagnosis of a PEComa (perivascular epithelioid cell tumour). The patient did not have a history of tuberous sclerosis. Surgery was decided at a multidisciplinary team meeting. During the procedure, we found a mass developed from the round ligament of the liver with numerous varicose veins. A mini laparotomy centered around the umbilicus was performed, providing adequate access to the lesion. No distant lesions were identified. There were no adhesions with the mesentery or the small bowel. However, its close association with multiple varicose veins required careful ligation to control venous drainage and prevent bleeding. Finally, ligation of the round ligament allowed for complete release and successful resection of the tumour, ensuring no further interference with surrounding structures. No bowel resection was necessary. Post‐operative course was uneventful, and patient was discharged on day 2. Histological analysis confirmed a PEComa measuring 10 cm in long axis. The immunohistochemical profile of the tumour cells was as follows: Smooth Muscle Actin (SMA)+, Desmin−, Caldesmon+, HMB45+, Melan‐A+, P53 WT, RB preserved, TFE3+, CK AE1/AE3−, S100−, and SOX10−. The low proliferative index (Ki67 < 10%) further supports the diagnosis. No adjuvant treatment was administered. Additionally, after 2 years of follow‐up, no recurrence has been detected (Figs. 1, 2, 3).

**Fig. 1:**
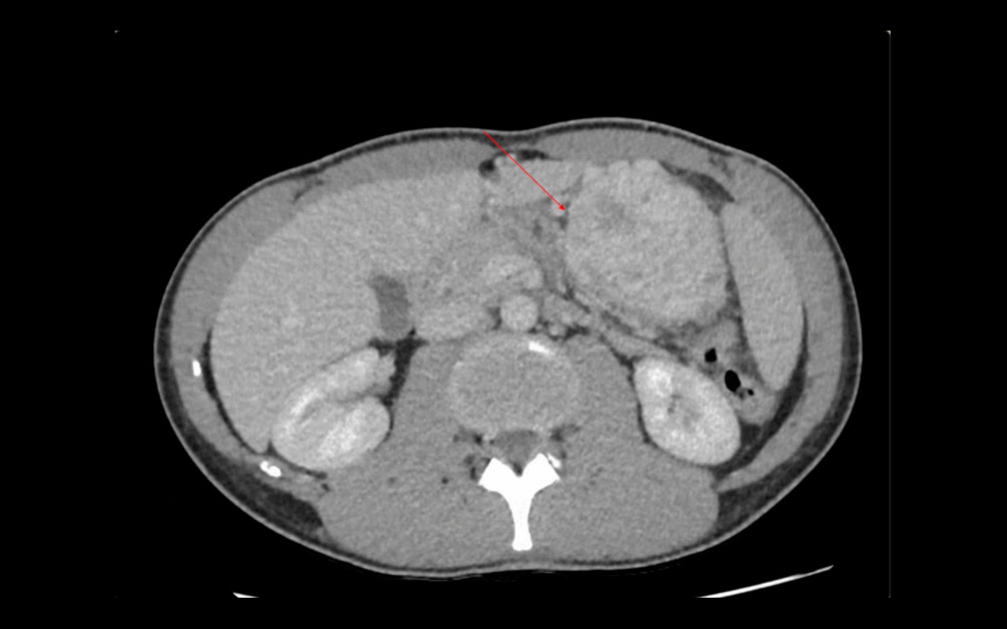
Computed tomography scan showing the intra‐abdominal lesion.

**Fig. 2:**
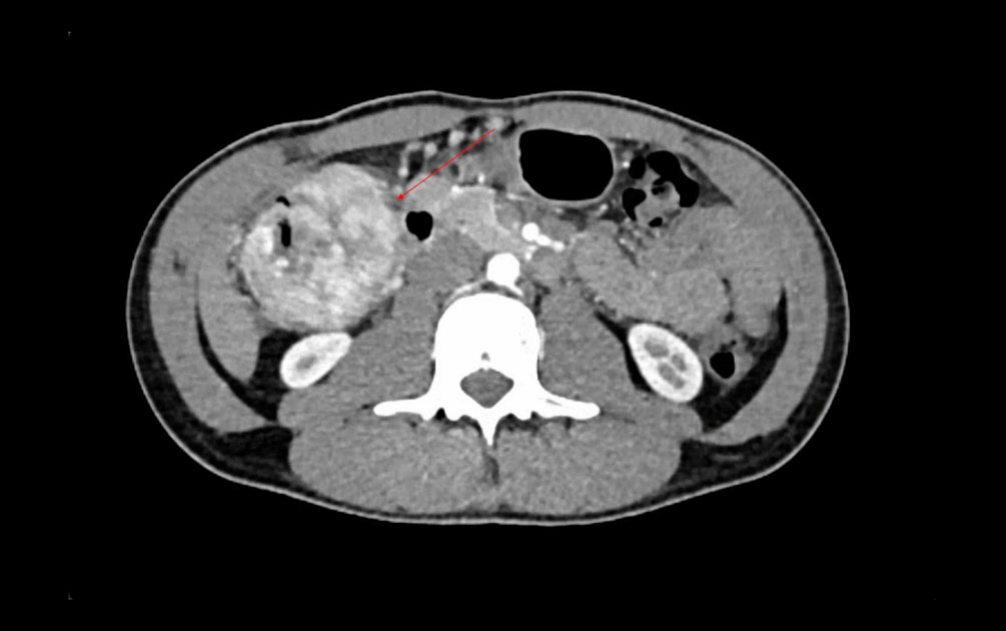
Computed tomography scan performed 1 month later demonstrating mobility of the lesion.

**Fig. 3:**
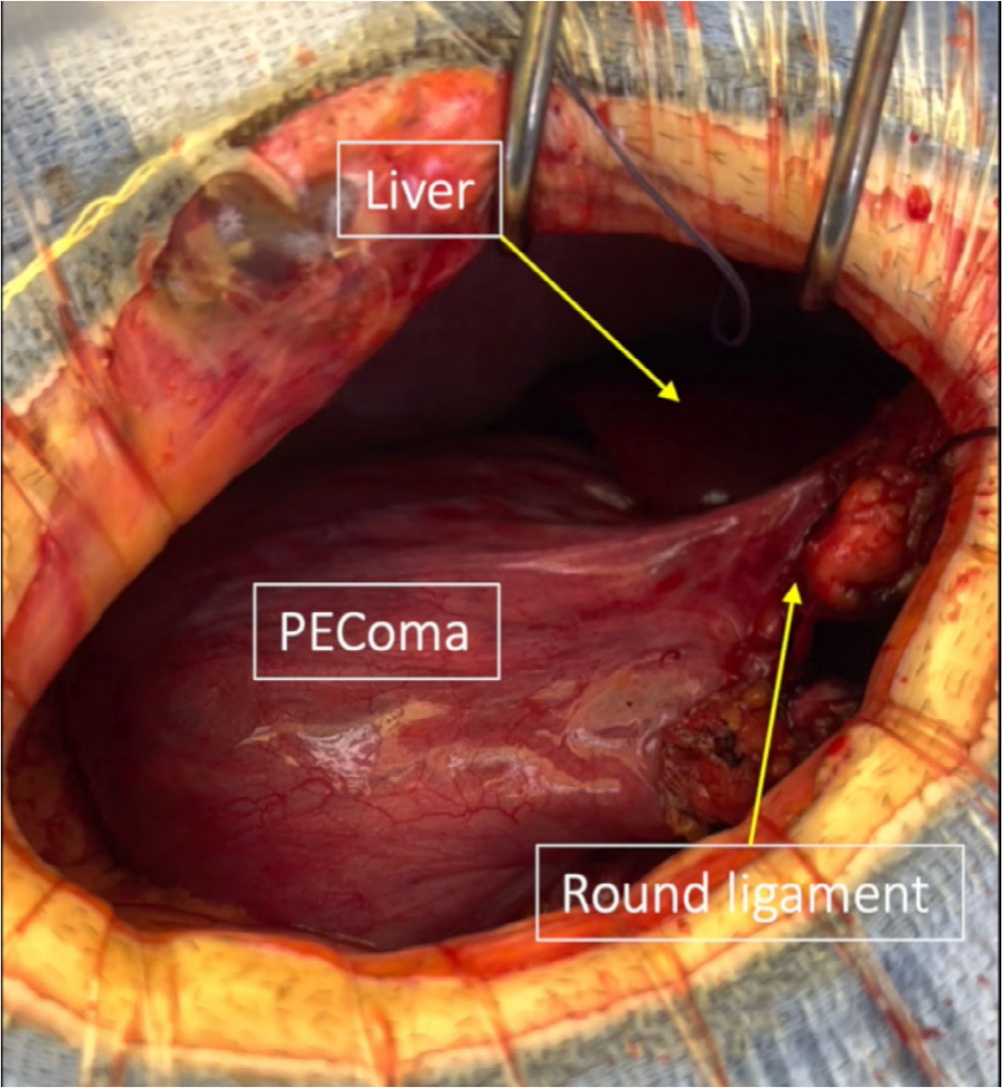
Intraoperative view.

The World Health Organization characterized PEComa as a tumour of mesenchymal origin that typically exhibits a close relationship with blood vessel walls and generally displays markers associated with melanocytes and smooth muscle cells.^1^, ^2^ Due to its extreme rarity, diagnosing PEComa is particularly challenging, with a significant potential for confusion with other soft tissue lesions. The preoperative differential diagnosis encompasses teratomas, leiomyosarcomas or gastrointestinal stromal tumours. This rare entity predominantly affects females^3^ and has been reported in various anatomical locations, including the uterus, liver, rectum, heart, breast, common bile duct, urinary bladder, abdominal wall, and pancreas.^1^, ^4^, ^5^ To the best of our knowledge, only 10 cases of PEComa arising from the ligamentum teres hepatis or the falciform ligament have been documented in the literature.^3^, ^6^, ^7^ This lesion is most observed in young women (<30 years old) and is occasionally associated with tuberous sclerosis.

Primary management for localized disease involves surgical resection. In a limited number of cases, neoadjuvant therapy has been employed with the sole aim of converting unresectable tumours into resectable ones.

Given the rarity of PEComa arising from ligamentum teres hepatis or falciform ligament, further studies and case reports are crucial to better understand its clinical behaviour, optimal diagnostic approaches, and therapeutic strategies. Multi‐institutional studies and comprehensive databases could standardize tumour management.

## Author contributions

**Jacques‐Emmanuel Saadoun:** Conceptualization; data curation; writing – review and editing. **Eddy Traversari:** Conceptualization; data curation. **Hélène Meillat:** Writing – review and editing. **Jérôme Guiramand:** Conceptualization; data curation; supervision; writing – review and editing.

## Supporting information


**Supplementary Video 1** Dynamic imaging of the perivascular epithelioid cell tumour (PEComa) of the ligamentum teres hepatis showing its unusual mobility during surgical manipulation.
